# Assessment of community support for *Wolbachia*-mediated population suppression as a control method for *Aedes aegypti* mosquitoes in a community cohort in Puerto Rico

**DOI:** 10.1371/journal.pntd.0009966

**Published:** 2021-12-06

**Authors:** Liliana Sánchez-González, Laura E. Adams, Rafael Saavedra, Emma M. Little, Nicole A. Medina, Chelsea G. Major, Marina Bruck, Julieanne Miranda, Coral Rosado-Santiago, Kyle Ryff, Marianyoly Ortiz, Grayson Brown, Roberto Barrera, Carmen L. Pérez-Guerra, Vanessa Rivera-Amill, Gabriela Paz-Bailey

**Affiliations:** 1 CDC Division of Vector Borne Diseases, Dengue Branch, San Juan, Puerto Rico; 2 Puerto Rico Vector Control Unit, San Juan, Puerto Rico; 3 Emory University, Atlanta, Georgia, United States of America; 4 Ponce Health Sciences University/Ponce Research Institute, Ponce, Puerto Rico; USDA-ARS Center for Medical Agricultural and Veterinary Entomology, UNITED STATES

## Abstract

Arboviral diseases transmitted by *Aedes* species mosquitoes pose an increasing public health challenge in tropical regions. *Wolbachia-*mediated population suppression (*Wolbachia* suppression) is a vector control method used to reduce *Aedes* mosquito populations by introducing male mosquitoes infected with *Wolbachia*, a naturally occurring endosymbiotic bacterium. When *Wolbachia-*infected male mosquitoes mate with female wild mosquitoes, the resulting eggs will not hatch. Public support is vital to the successful implementation and sustainability of vector control interventions. Communities Organized to Prevent Arboviruses (COPA) is a cohort study to determine the incidence of arboviral disease in Ponce, Puerto Rico and evaluate vector control methods. Focus groups were conducted with residents of COPA communities to gather their opinion on vector control methods; during 2018–2019, adult COPA participants were interviewed regarding their views on *Wolbachia* suppression; and a follow-up questionnaire was conducted among a subset of participants and non-participants residing in COPA communities. We analyzed factors associated with support for this method. Among 1,528 participants in the baseline survey, median age was 37 years and 63% were female. A total of 1,032 (68%) respondents supported *Wolbachia* suppression. Respondents with an income of $40,000 or more were 1.34 times as likely [95% CI: 1.03, 1.37] to support *Wolbachia* suppression than those who earned less than $40,000 annually. Respondents who reported repellant use were 1.19 times as likely to support *Wolbachia* suppression [95% CI: 1.03, 1.37]. A follow-up survey in 2020 showed that most COPA participants (86%) and non-participants living in COPA communities (84%) supported *Wolbachia* suppression during and after an educational campaign. The most frequent questions regarding this method were related to its impact on human and animal health, and the environment. Continuous community engagement and education efforts before and during the implementation of novel vector control interventions are necessary to increase and maintain community support.

## Author summary

Vector control is key to decrease the incidence of viral infections transmitted by *Aedes* mosquitoes, like dengue, but traditional control methods like insecticides and breeding sites reduction can be time-consuming, ineffective, and challenged by insecticide resistance. In the method known as *Wolbachia* suppression, male mosquitoes infected with the bacterium *Wolbachia* are released to mate with wild female mosquitoes. As the resulting eggs do not hatch, the mosquito population decreases over time. As public support is key to implement and maintain any vector control method, we assessed community support for *Wolbachia* suppression through focus groups and interviews with residents of Ponce, Puerto Rico, where a community cohort has been implemented. Support for this method in this population was high and increased after an educational campaign. Respondents with higher income and those who used repellent in the last 30 days were more likely to support *Wolbachia* suppression. Questions regarding the impact of this method on human and animal health, and the environment, were the most reported. Community engagement activities to increase the understanding of the method, including clear and specific information on health risk assessments, should be conducted before and during the implementation of *Wolbachia* suppression.

## Introduction

Dengue viruses (DENV) and other arboviruses transmitted by *Aedes* species mosquitoes, including Zika (ZIKV) and chikungunya (CHIKV), pose increasing public health challenges in tropical regions [1,2]. About half the global population is estimated to live in areas at risk of dengue transmission, with most in Asia, followed by Africa and the Americas [3,4]. Most dengue infections are asymptomatic, but some patients may progress to severe dengue, which is characterized by increased vascular permeability and can be fatal [5]. Dengue is endemic in Puerto Rico, where epidemics typically follow a cyclical pattern occurring about every five years [6], with the most recent outbreaks occurring in 2010 and 2012–2013 [7,8]. More than 18,000 suspected cases, 9,200 confirmed cases, and 12 deaths were reported during the last epidemic [9]. CHIKV and ZIKV have caused major global epidemics in the past decade in many areas also affected by DENV [10–12]. Puerto Rico experienced a CHIKV outbreak in 2014 with more than 4,500 confirmed cases reported, followed by a ZIKV outbreak in 2016 with more than 36,000 confirmed cases reported [13–16]. The long-term sequelae of these viruses have caused considerable economic impact and burden to the healthcare system during and following outbreaks.

Historically, *Aedes* vector control has relied heavily on synthetic insecticides and environmental management of breeding sites [17]. Environmental management, which entails identifying, reducing, and removing mosquito breeding sites can be time-consuming and inefficient. High levels of insecticide use, including DDT, pyrethroids, carbamates, and organophosphates, increase the risk of selecting for insecticide-resistant alleles and creating populations of resistant vectors [18,19]. Resistance to the four main classes of insecticides has been detected in *Aedes* mosquito populations across the Americas, Asia, and Africa [20]. In Puerto Rico, widespread resistance of *Aedes aegypti* mosquitoes to pyrethroids has been documented, with geographical heterogeneity further hindering the use of insecticides for vector control on the island [21,22]. In addition, low confidence in and acceptance of insecticides reported in dengue-endemic communities pose a further barrier to their use [23].

In the search for sustainable and effective alternatives to insecticides, *Wolbachia pipientis*, an intracellular endosymbiotic bacterium found in about 60% of all insects [24] but not commonly found in wild *Aedes aegypti* populations [25], has been studied and used as a novel strategy for dengue control. In an approach known as *Wolbachia* replacement, *Wolbachia*-infected male and female mosquitoes, which have a reduced capacity to transmit arboviruses, are released to replace a wild population of *Aedes* mosquitoes [26,27]. This bacterium can also be used to reduce the wild population of *Aedes* mosquitoes, an approach known as *Wolbachia-*mediated population suppression, hereafter referred to as *Wolbachia* suppression [28].

In the *Wolbachia* suppression method, *Wolbachia*-infected *Aedes* males are released into the environment to mate with wild females. The resultant eggs will not hatch due to cytoplasmic incompatibility, reducing the wild mosquito population’s fecundity and size [29]. This technique can effectively reduce the population over time while *Wolbachia*-infected male mosquitoes continue to be released and has demonstrated suppression levels above 80% in *Aedes aegypti* populations [30,31].

The Communities Organized to Prevent Arboviruses (COPA) project is an ongoing community cohort study initiated in early 2018 to better understand, prevent, and control diseases spread by *Aedes aegypti* mosquitoes in Ponce, a municipality with historically high levels of arboviral diseases in Puerto Rico. COPA is a collaboration between the Ponce Health Sciences University/Ponce Research Institute, the Puerto Rico Vector Control Unit (PRVCU), and the Centers for Disease Control and Prevention (CDC). The objectives of the project include evaluation of the impact of *Wolbachia* suppression method on decreasing *Aedes aegypti* populations and in preventing arboviral infections in humans, assessed through annual sero-surveys.

Reduction in entomological and human disease outcomes has been previously associated with high levels of community buy-in in cluster randomized trials for a variety of vector control interventions [32]. As a novel vector control strategy that requires the *Wolbachia* infected mosquitoes to be released into the community, awareness and support of involved communities are critical to this program’s success. Although there is information on knowledge, attitudes, and practices regarding dengue infection in Puerto Rico [33–35], data is very limited regarding novel vector control interventions acceptability and the factors determining community support.

We present data from qualitative interviews during formative research, the baseline survey among adult participants in COPA study clusters, and a follow-up survey among a subset of COPA participants and study cluster residents not participating in the cohort. We analyzed levels of support for different vector control strategies and the association of *Wolbachia* suppression support with key demographic and behavioral variables.

## Methods

### Ethics statement

The three studies were reviewed by the CDC Human Subjects Office and approved by the Ponce Medical School Foundation, Inc. Institutional Review Board, approval number 171110-VR. COPA participants provided written consent for the 2018–2019 baseline interview. COPA cohort participants and non-participants provided verbal assent for the 2017–2018 formative research activities and for the 2020 follow-up survey.

**Fig 1:**
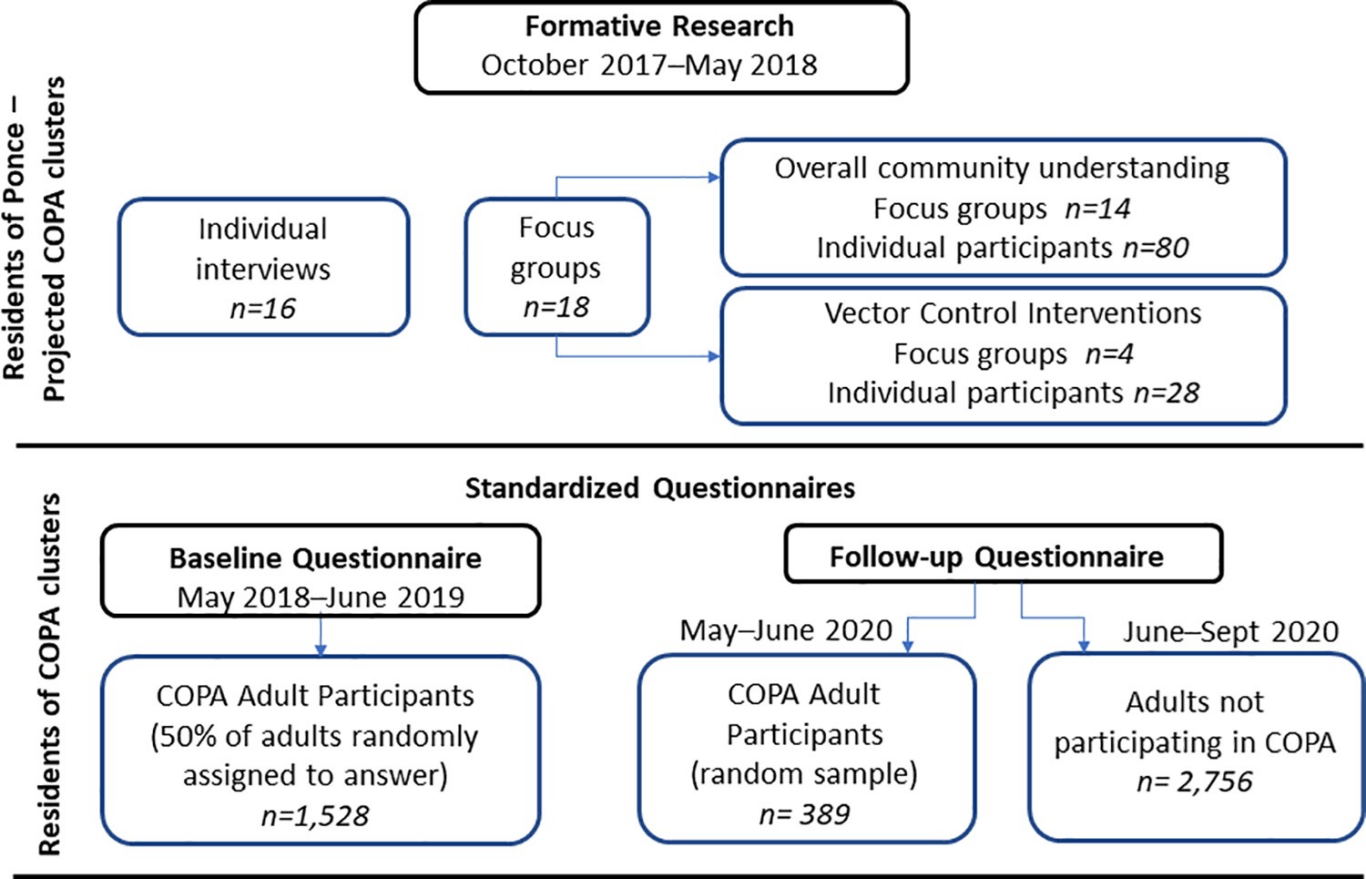
Summary of data sources for assessment of community acceptability of *Wolbachia* suppression method, Ponce, Puerto Rico, 2017–2020.

Data from these analyses came from three sources: 1) qualitative interviews and focus groups with selected Ponce community leaders and residents in 2017–2018, 2) baseline data from 2018–2019 COPA cohort participants in 38 study clusters with a high historic incidence of dengue, chikungunya, and Zika in the municipality of Ponce, Puerto Rico, and 3) follow-up data from 2020 collected from a random sample of COPA cohort participants through phone interviews and a sample of non-participating study cluster residents identified and interviewed in house-to-house visits (Fig 1).

### Formative research

During October 2017–May 2018, the CDC Dengue Branch behavioral health and communications team conducted informal interviews, focus group discussions, and in-depth interviews with adult (>21 years old) residents and community leaders in the projected COPA clusters (delimitation of clusters had not been finalized at the time) to better understand the organization of communities participating in the project, identify the main perceived problems in the community, and assess attitudes and perceptions towards mosquito-borne diseases and six vector control methods: autocidal gravid ovitraps (AGO), larvicides, indoor residual spraying (IRS), *Wolbachia* suppression (release of male mosquitoes with *Wolbachia*), *Wolbachia* replacement (release of male and female mosquitoes with *Wolbachia*) and genetically modified (GM) mosquitoes. Before the interviews and focus groups, no specific information on any of the techniques was provided to participants, they were only informed of the overall theme of the activities.

The researchers used the “snowball” technique [36]: the team spoke with a handful of their existing contacts in the communities of interest, known from previous projects. They were invited to participate in this project, and then were asked to help identify their community leaders and other community residents who, in turn, identified other potential participants and their community leaders, and so on. The researchers then made phone calls or visited potential participants at their homes to invite them to the focus group sessions. In-depth interviews were conducted with community leaders also identified with the “snowball” technique; leaders who participated in the focus groups identified other leaders to participate in in-depth interviews. Participation in all formative research activities was voluntary, and no demographic or other identifiable information was collected from participants.

### COPA cohort baseline survey

Within the 38 defined study clusters, the COPA study staff recruited participants in randomly selected households using ArcGIS. Selected households were visited up to three times on different days to offer enrollment, including at least one Saturday. Study participation was offered to all household members in selected households who were aged 1–50 years old, slept in the house at least four nights per week, and did not have plans to move in the next six months.

Information applicable to all household members, including annual household income, water source, and use of screens and air conditioning, was obtained from one representative per household. In addition, a questionnaire capturing information about demographics, history of febrile illness in the last year, and personal protective behaviors related to mosquito avoidance, prevention, and control was administered to all participants. We also asked about perceived risk and burden of arboviral disease in the community. COPA cohort participants are defined as those who answered the study annual questionnaires and provided serum samples for arboviral testing.

For the assessment of the acceptability of vector control methods, half of the eligible adult participants aged 21–50 were randomly selected to participate in the Vector Control Interventions (VCI) questionnaire. Participants under 21 were not eligible for this component. Interviewers read a script describing six different mosquito control interventions (S1 Appendix), and asked participants if they would support or oppose the use of the following interventions in their community: autocidal gravid ovitraps (AGO), larvicides, indoor residual spraying (IRS), *Wolbachia* suppression (release of male mosquitoes with *Wolbachia*), *Wolbachia* replacement (release of male and female mosquitoes with *Wolbachia*) and genetically modified (GM) mosquitoes.

After six months of data collection, we removed three interventions from the survey (IRS, AGO, *Wolbachia* replacement) because it was determined that those interventions were unlikely to be implemented, given logistics (availability of effective insecticides for IRS and large coverage area for AGO) and regulatory challenges (*Wolbachia* replacement does not currently have FDA approval in the United States or its territories), and *Wolbachia* suppression was the intervention chosen to be implemented. We evaluated differences in the level of support for *Wolbachia* suppression by participant demographics and mosquito knowledge and avoidance practices.

### Follow-up survey

We assessed the level of support for *Wolbachia* suppression from COPA participants and a selection of non-participating residents in the study clusters as a follow-up for the baseline data in two phases during 2020. In Phase 1 (May–June 2020), we selected COPA participants to contact for phone interviews with a goal of 10 participants in each cluster (N = 380). In Phase 2 (June–September 2020), we conducted surveys among 2,756 non-participating residents of study clusters during house visits. Phase 2 occurred during and following an educational media campaign on *Wolbachia* suppression that was implemented by PRVCU. Both Phase 1 and Phase 2 utilized a survey asking whether respondents had heard of *Wolbachia* suppression, and then received information about how approach works and its safety and efficacy via a short video or a corresponding standardized script read by the interviewer. After the educational component, residents were asked if they had any questions about the *Wolbachia* suppression approach for mosquito control, which were answered by trained interviewers using supplementary materials. Residents were then asked if they had heard about the use of *Wolbachia* suppression in Puerto Rico and any sources of information they had used to learn about this vector control method. Finally, residents were asked if they supported, were neutral, or opposed the use of *Wolbachia* suppression in Puerto Rico and in their community. Participants that opposed *Wolbachia* suppression were asked to explain their reasons.

### Analyses

For the COPA baseline data, we summarized participant demographics, personal protective behaviors, and household-level factors that could affect arboviral infection risk. We calculated relative risks (RR) and 95% confidence intervals (CI) using Cochran-Mantel-Haenszel statistics to assess variables associated with *Wolbachia* suppression support.

For the follow-up survey data, we included descriptive analyses to report on demographic characteristics, risk perception, and *Wolbachia* suppression acceptability. Chi-square analysis was performed to determine if there was difference in the *Wolbachia* suppression acceptance between COPA participants and non-participating residents of the 38 COPA clusters. A p value <0.05 was considered significant.

## Results

### Formative research

We conducted 14 focus groups with residents and community leaders (n = 80) in addition to 16 individual interviews with community leaders to better characterize and understand their communities. Participants were asked what they considered the most pressing issues in their community. Community leaders reported that before Hurricane Maria (September 2017), the most important problems were related to infrastructure, such as deteriorated roads and buildings; health issues such as mosquito-borne diseases and asthma; and social issues such as crime, drug use, and interpersonal conflict. Focus group members shared that after the hurricane, debris and trash removal and municipal mosquito spraying notably decreased or were absent, noting problems with standing water and mosquitoes. Participants observed that the increased amount of trash attracted rats and there were concerned about cases of leptospirosis. Already weak public works activity, resulting in clogged sewers, flooding, broken pipes, lack of weeding, clandestine landfills, and overgrown trees was perceived to have worsened after Hurricane Maria.

*“We are talking with the municipality to see if they will help us with that. … People throw garbage here into the water canal. Well, that would affect mosquito and other insect breeding sites… I’m telling you, this is a mosquito warehouse."*

To prevent mosquito bites, participants most frequently reported spraying insecticides and using area repellants including bonfires, citronella candles, insecticide spirals, and homemade methods. The leaders also reported using protective clothing, screens, fans, mosquito nets, and avoiding being outside the house to prevent mosquito bites. To reduce mosquito breeding sites in the home, participants reported emptying containers that collect water, cutting the grass, and keeping the surroundings clean. Some residents reported setting mosquito traps, including homemade traps. In addition, some focus groups members mentioned they stay informed with educational materials and by attending health talks.

During 4 additional focus groups discussing specific mosquito vector control strategies, only 2 out of 28 participants (7%) had heard about Wolbachia suppression method before the groups, and 18 participants (64%) reported they were supportive of the use of *Wolbachia* suppression in their communities. After explaining all six methods, a respondent preferred *Wolbachia* suppression over *Wolbachia* replacement because *“it is better if mosquito eggs don’t hatch”*. Another participant preferred *Wolbachia* suppression over other methods because a perceived advantage of reaching all wild mosquitoes, the method *“…will not eliminate some and not others…it will take them all”*. Participants also voiced concerns about implementing any technique that *“comes from a laboratory”* in their communities, considered the need to continue releases to keep mosquito populations suppressed a disadvantage of the *Wolbachia* suppression method, and some participants voiced their preference for more traditional methods like insecticide spraying and requested more information on results of the technique in other geographical areas. The results of the formative research were shared with COPA study collaborators to help inform the study activities, including the development of standardized questionnaires and informational and educational campaigns and materials.

### COPA cohort baseline survey

A total of 4,724 participants were enrolled during the project baseline in 2018–2019, of which 1,528 (32%) answered the vector control interventions questions. Among them, median age was 37 years [IQR: 28–44], and 962 (63%) were female. A total of 972 (64%) participants reported an education level below a bachelor’s degree, and 858 (56%) participants reported an annual household income of less than $20,000 (Table 1).

**Table 1:** Characteristics of study participants responding to the Vector Control Interventions survey, COPA, Ponce, Puerto Rico 2018–2019.

Characteristic	N (%)
Total	1,528
**Sex**	
Women	962 (63.0)
**Age**	
21–30	466 (30.5)
31–40	469 (30.7)
41–50	593 (38.8)
**Education Level** *	
Lower Education	972 (63.6)
Higher Education	542 (35.5)
**Annual Income**	
Less than $20,000	858 (56.5)
Less than $40,000	358 (23.4)()
$40,000 or Above	220 (14.4)
**Employment Environment (n = 716)**	
Primarily Indoors	537 (66.1)
Primarily Outdoors/ Varied	275 (33.9)
**Believe Mosquitoes Transmit Disease**	1,436 (94.0)
**Perceived Risk of Getting a Disease from Mosquitoes**	
High	353 (23.1)
None/Low	817 (53.5)
No response	358 (23.4)
**Perception of Arboviruses as an Issue in the Community**	
Perceive Arboviruses as an Issue in Community	997 (65.3)
**Expenditure of Time and Money for Vector Control**	
Believe it is Worth Investing Time and Money for Vector Control	1,451 (95.0)
**Annual Household Expenditure on Mosquito Control**	
Less than $120	535 (35.0)
$120 or Above	901 (59.0)
No response	92 (6.0)
**Personal Repellant Use in Past 30 Days**	
Have Used Repellant in Past 30 Days	840 (55.0)
**Use of Mosquito Net in Past Year**	
Have Used Mosquito Net in Past Year	30 (2.0)
**Role of Government/ Department of Health in Vector Control**	
Believe Government/Dept of Health Should Have Some Responsibility in Vector Control	1,258 (82.3)
**Have Previously Heard of Mosquitoes with *Wolbachia***	71 (4.7)

*Lower education was defined as completing up to a technical degree or associate degree. Higher education was defined as completing a bachelor’s degree, professional degree, or post-graduate studies.

Regarding perceptions and prevention behaviors, 1,451 participants (95% of those who answered the question) thought it was worth investing in vector control, 1,258 (82%) believed the government and health department had responsibility in vector control, 997 (65%) perceived arboviruses were an issue in the community, and 353 (43%) perceived that they had a high risk of getting a disease from mosquitoes. Overall, 840 (55%) reported using mosquito repellant in the past 30 days and 30 (2%) reported they had used a mosquito net in the last year (Table 1).

**Fig 2:**
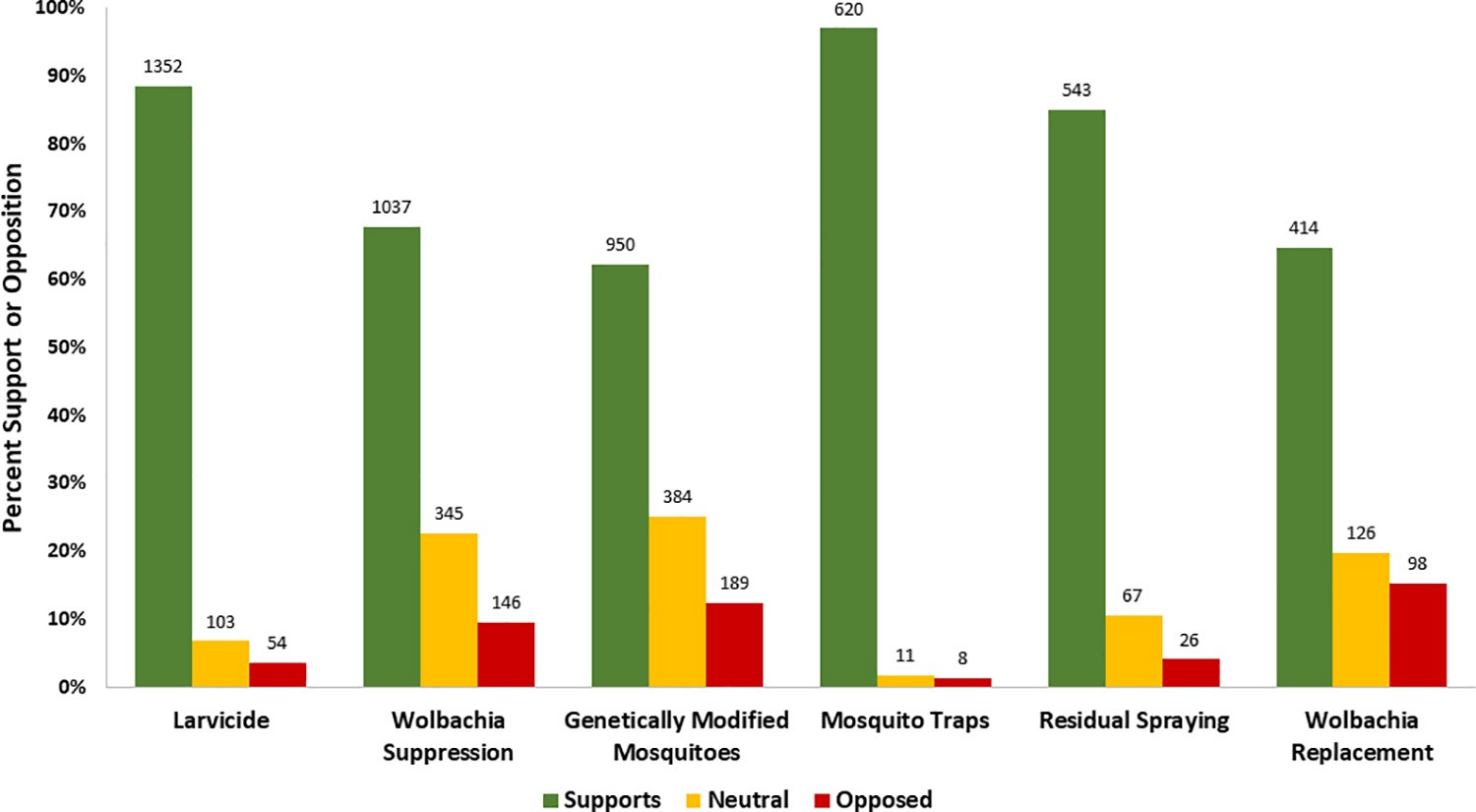
Acceptability of vector control interventions, COPA, Ponce, Puerto Rico, 2018–2019.

Among participants who answered questions for larvicides, *Wolbachia* suppression, and genetically modified mosquitoes, 1,352/1,509 (88%), 1,037/1,528 (67%), and 950/1,523 (62%) supported each technique, respectively (Fig 2). Among 639 participants who answered questions on AGO traps, indoor residual spraying, and *Wolbachia* replacement, 620 (97%), 543 (85%), and 414 (65%) supported each technique, respectively.

A minority of participants (71; 5%) had heard of mosquitoes with *Wolbachia* for either suppression or replacement methods before the interview. Those with an annual income of $40,000 or more were more likely to support *Wolbachia* suppression (RR: 1.34, 95% CI: 1.04, 1.73) than those with an income under $20,000 (Table 2). There were no differences in *Wolbachia* suppression support among respondents by sex, age, or educational level.

**Table 2:** Factors associated with *Wolbachia* suppression support, COPA, Ponce, Puerto Rico, 2018–2019.

Variable	Acceptance of *Wolbachia s*uppression N (%)	Total N	RR	95% CI
**Total**	**1,037 (67.9%)**	**1,528**		
**Sex**				
Men^r^	392 (69.3)	566		
Women	645 (67.1)	962	1.07	0.92, 1.25
**Age**				
21–30^r^	323 (69.3)	466		
31–40	321 (68.4)	469	0.97	0.80, 1.18
41–50	393 (66.3)	593	0.91	0.76, 1.09
**Education Level** ^**a**^				
Lower Education ^r^	656 (67.5)	972		
Higher Education	370 (68.3)	542	1.01	0.94, 1.09
**Annual Income**				
Less than $20,000^r^	581 (67.7)	858		
20,000 to $39,999	234(65.4)	358	0.93	0.78, 1.11
$40,000 or Above	167 (75.9)	220	**1.34**	**1.04, 1.73**
**Employment Environment** ^**b**^				
Primarily Indoors^r^	343 (68.1)	504		
Primarily Outdoors/Varied	151 (68.3)	221	1.01	0.80, 1.27
**Believe Mosquitoes Transmit Disease**				
No^r^	45 (59.2)	76		
Yes	983 (68.5)	1,436	1.29	0.98, 1.71
**Perceived Risk of Getting a Disease from a Mosquito**				
None/Low^r^	558 (68.3)	817		
High	249 (70.5)	353	1.03	0.95, 1.12
**Perceive Arboviruses as a Problem in the Community**				
No^r^	298 (66.4)	449		
Yes	688 (69.0)	997	1.09	0.93, 1.27
**Believe it is Worth Investing Time and Money for Vector Control**				
No^r^	46 (68.7)	67		
Yes	986 (68.0)	1,451	0.98	0.68, 1.41
**Annual Household Expenditure on Mosquito Control**				
Less than $120^r^	343 (64.1)	535		
$120 or Above	638 (70.8)	901	1.10	1.02, 1.19
**Have Used Repellant in Past 30 Days**				
No^r^	442 (64.8)	682		
Yes	591 (70.4)	840	**1.19**	**1.03, 1.37**
**Have Used Mosquito Net in Past Year**				
No^r^	1,011 (68.1)	1,485		
Yes	17 (56.7)	30	0.83	0.61, 1.14
**Believe Government/Dept of Health Should Have Some Responsibility in Vector Control**				
No^r^	168 (72.4)	232		
Yes	849 (67.5)	1,258	0.85	0.68, 1.06
**Have Previously Heard of Mosquitoes with *Wolbachia***				
Never/Unsure^r^	987 (67.7)	1,457		
Yes	50 (70.4)	71	1.09	0.76, 1.57

^**r**^ = Reference Group

^a^ = Group Includes Special Education, No Education, Grades 1–11, Completed High School/GED, and those with a Technical or Associate Degree

^b^ = Group Only Includes Individuals Who Responded to Employment Status with "Full time", "Part-time", "Student" or "Work/Study"

There were also no differences in *Wolbachia* suppression support by arbovirus risk perception, knowledge that mosquitoes are vectors of diseases, perception of mosquitoes as a community problem, annual expenditure on mosquito control, use of mosquito nets, or previous knowledge of mosquitos with *Wolbachia*. Among 840 participants who reported using repellant in the last 30 days, 70% supported *Wolbachia* suppression compared to 65% of those who reported no use of repellant in the past 30 days (RR: 1.19, 95% CI: 1.03, 1.37).

The support for *Wolbachia* suppression in the 38 study clusters ranged from 42% to 88%, with most clusters (n = 28, 74%) reporting *Wolbachia* suppression support levels between 50% and 75% (Table 3).

**Table 3:** *Wolbachia* Suppression support by cluster, COPA cohort baseline survey, Ponce, Puerto Rico, 2018–2019.

CLUSTER	Total interviewed	Supports (n, %)	CLUSTER	Total interviewed	Supports (n, %)
COPA01	26	11 (42%)	COPA20	38	26 (68%)
COPA02	59	31 (53%)	COPA21	16	11 (69%)
COPA03	45	24 (53%)	COPA22	32	22 (69%)
COPA04	30	17 (57%)	COPA23	20	14 (70%)
COPA05	21	12 (57%)	COPA24	30	21 (70%)
COPA06	24	14 (58%)	COPA25	31	22 (71%)
COPA07	33	20 (61%)	COPA26	94	68 (72%)
COPA08	46	28 (61%)	COPA27	58	42 (72%)
COPA09	39	24 (62%)	COPA28	92	67 (73%)
COPA10	39	25 (64%)	COPA29	30	22 (73%)
COPA11	31	20 (65%)	COPA30	29	22 (76%)
COPA12	26	17 (65%)	COPA31	25	19 (76%)
COPA13	29	19 (66%)	COPA32	46	35 (76%)
COPA14	35	23 (66%)	COPA33	39	30 (77%)
COPA15	41	27 (66%)	COPA34	28	22 (79%)
COPA16	21	14 (67%)	COPA35	34	27 (79%)
COPA17	39	26 (67%)	COPA36	20	16 (80%)
COPA18	50	34 (68%)	COPA37	36	29 (81%)
COPA19	22	15 (68%)	COPA38	24	21 (88%)

### Follow-up survey among COPA participants and cluster residents

A total of 389 COPA participants from the 38 study clusters (range: 4–15 participants per cluster) responded to a follow-up phone interview in 2020. Median respondent age was 42 years (IQR: 33–48), 263 (68%) were female, 232 (60%) reported an education level below a bachelor’s degree, and 225 (83% of those with income data) reported an annual household income <$40,000. A total of 103 (26%) participants reported they had heard of male mosquitoes with *Wolbachia* before the interview and the same percentage said they knew the technique would be used in Puerto Rico. A total of 68 (17%) had questions about the use of this technique; 14 (21%) of which were related to its impact on human and animal health and the environment, and 24 (35%) about the technique itself and its use in Puerto Rico (Table 4). Overall, 333 (86%) COPA participants supported the use of *Wolbachia* suppression in Puerto Rico during the follow-up interviews, 13 (3%) were opposed and 43 (11%) were neutral or did not know.

**Table 4:** Participants’ questions regarding the use of male mosquitoes with *Wolbachia* during follow-up survey, COPA, Ponce, Puerto Rico 2018–2019.

Theme*	Phase I COPA participants May-Jun 2020		Phase II Non-participants Jun-Sep 2020	
	n	%	n	%
The use of the technique in Puerto Rico	11	16.2	114	32.3
Impact on human health	14	20.6	103	29.2
Environmental impact	5	7.3	68	19.3
Development	7	10.3	61	17.3
Mosquito production	6	8.8	58	16.4
Impact on animals/domestic animals	6	8.8	33	9.3
The use of the technique in other places	1	1.5	31	8.8
Cost	4	5.9	8	2.3
Approval	3	4.4	7	1.9
Other	43	66.2	78	22.1

*Answers are not exclusive. One participant could select and ask different questions.

For the interviews of non-participant residents of COPA clusters, 7,535 houses were visited in the 38 clusters. Contact was made with 2,885 (38%) households and 2,756 residents were interviewed. No sociodemographic data were collected among non-participants. About half (1,349, 49%) of residents interviewed had heard of the use of mosquitoes with *Wolbachia* before the visit and were aware of its possible use in Puerto Rico. A total of 353 (13%) respondents had questions about different aspects of the technique itself, 114 (32%) had questions on how this technique would be used in Puerto Rico, and 103 (29%) had questions on the impact on human health, animal health, and the environment (Table 4). Support for *Wolbachia* suppression among non-participant residents of COPA clusters in their community was 84% (n = 2,328), with 4% (n = 122) opposed and 306 (11%) neutral or unsure.

There was no significant difference in the acceptance of *Wolbachia* suppression between COPA participants and non-participants in the study clusters (p = 0.957).

## Discussion

This study found a high level of support for six vector control methods presented to participants during formative research and baseline and follow-up interviews. Traps, larvicides, and indoor residual spraying had a higher level of acceptance than *Wolbachia* methods or GM mosquitoes. During formative research and baseline, two- thirds of participants supported the use of *Wolbachia* suppression in their communities, despite a small number having heard of it previously, with main concerns being related to this technology being developed in a laboratory and the results of the use of the technique in other places. Participants with higher income and those who reported using repellant in the last 30 days were more likely to accept *Wolbachia* suppression. Overall, the baseline risk perception for dengue infection in this population was low (23%), and the majority of participants believed the government is responsible for vector control. During follow-up with COPA participants, the proportion of participants who had heard of *Wolbachia* increased five-fold, and similarly high levels of support (85%) and low levels of opposition were reported among all residents of COPA clusters. These findings likely reflect the impact of educational and informational campaigns developed and implemented between the initial assessment and follow up.

Multiple factors have been associated with community support, opposition, and participation in different vector control interventions. The occurrence of natural disasters and epidemics can influence risk perception and support for interventions. A survey in the United States Virgin Islands found that acceptance of some mosquito control and prevention activities was higher during an active hurricane response than during a previous survey during a Zika outbreak [37]. The formative research we conducted in COPA after Hurricane Maria found increased concerns among community members about mosquito-borne diseases and a sense of urgency to implement control measures, which can influence the acceptance of novel vector control interventions. As *Aedes aegypti* control has proven to be very challenging and communities continue to be affected by arboviral epidemics, communities are more open to accepting promising control strategies [38]. However, novel interventions included in this study (*Wolbachia*-based and GM mosquitoes) had lower support than other more traditional interventions, highlighting the importance of community engagement to increase understanding of novel vector control interventions. Low community awareness has been shown to be a barrier to effective community engagement on vector control [39], and community involvement in these programs can improve their effectiveness [40,41]. Although *Wolbachia* suppression is delivered as a vertical program, community awareness and understanding are essential to improve acceptance and ensure the uninterrupted delivery of the intervention.

We found that participants who reported using repellant in the last 30 days, an active personal preventive practice against mosquito bites, had higher levels of support for *Wolbachia* suppression. This can be explained by higher perceived personal risk perception among these participants. However, there was no difference in *Wolbachia* suppression acceptance by other related factors, including dengue risk perception, perception of mosquitoes as a community problem, expenditure on mosquito control, or awareness of mosquitoes as vectors for disease. A lack of correlation between risk perception and acceptability of vector control interventions has been reported before [42]. An additional consideration is that the baseline survey was conducted during a period of minimal arbovirus transmission on the island, likely further lowering risk perception.

Other factors that have been associated with vector control interventions acceptance include attitudes toward government-run vector control operations and opinions of vector control organizations [38,42]. The Puerto Rico Vector Control Unit is part of a private nonprofit organization that regularly interacts with COPA communities through educational activities and has a positive reputation on the island. Few participants had questions regarding the intervention costs, and this might be related to the fact that most participants believed government organizations are responsible for vector control, as has been found in previous surveys in Puerto Rico, the United States, and other countries [43–47].

Limited information is available regarding factors specifically associated with *Wolbachia* suppression acceptance in Puerto Rico as this is the first time the technique is used in the island. A recent study in Singapore, where *Wolbachia* suppression implementation started in 2016, found high levels of *Wolbachia* suppression acceptability, with over 80% of participants interviewed, before and after the implementation, supporting the use of the technique [48]. This study found males to be more likely to support the method, as well as those who considered dengue to be a serious problem in their communities. We did not find an association between demographic characteristics or arboviral diseases risk perception and *Wolbachia* suppression acceptability, which highlights the importance of using local data obtained from the communities to be impacted when assessing acceptance of vector control methods and developing strategies to improve the support of local communities. When additional existing or newly developed novel vector control interventions are deemed suitable to be implemented in Puerto Rico, additional assessments will be needed to determine the acceptability of those specific interventions.

The most common questions about *Wolbachia* suppression during the follow-up interviews were related to details of its use in Puerto Rico and its potential impact on human health and the environment. Educational campaigns and materials directed to the public developed by the PRVCU have highlighted these aspects and should continue to address areas where participants had concerns, including information on the development and production of mosquitoes with *Wolbachia*; the potential impact on humans, animals, and the environment; costs; and regulatory approvals in place.

There are several limitations to this study. First, study participants might not be representative of all community members, as people who refused to participate or were not eligible to participate in COPA might be less likely to support novel vector control interventions than participants. However, the follow-up survey included both participants and non-participants and found similar levels of support across the two groups. Additionally, many of the participants were previously unfamiliar with the new vector control interventions, and their level of support for the interventions could change as they learn more about the topic. Participants in this assessment only represented areas within the COPA study clusters, and findings might not be generalizable to the rest of Puerto Rico. This intervention is not intended to be implemented in the whole island (as of publication), and the data obtained will contribute to developing better messaging and community outreach in the same areas where the surveys were conducted. Finally, the survey results could be affected by social desirability bias, as participants might have reported a greater support for prevention methods thinking this corresponded with the “right answer”. The PRVCU’s involvement in the surveys’ administration could have positively influenced the participants’ answers, as they are considered as a trustworthy organization. However, we obtained different levels of support for different interventions during the baseline, ranging from 63% to 97%, which suggests participants felt comfortable reporting opposition to interventions they did not support.

Novel vector control methods have the potential to reduce the burden caused by arboviral diseases in Puerto Rico and other areas with *Aedes aegypti* mosquitoes. One key aspect to the implementation and success of any vector control program—particularly those which can be perceived by involved communities as novel or experimental—is building trust with communities and engaging stakeholders at all levels. Our results show that *Wolbachia* suppression has high acceptance in COPA study communities. To maintain public support and buy-in for effective implementation of *Wolbachia* suppression, it is essential to actively engage community members through regular educational activities, maintain an organizational presence in the communities, and to acknowledge and address community members concerns. Future assessment activities could include additional data collection on acceptability of the technique after completion of the project. Although we did not find a correlation between risk perception and *Wolbachia* acceptance, increasing awareness on the risk of infection with dengue and other arboviral diseases could increase prevention practices at the personal level and acceptance of vector control interventions at the community level. It is important to consider individual and community priorities and engage community leaders in designing and implementing outreach strategies for vector control interventions.

## Supporting information

S1 Appendix Vector Control Methods Questionnaire English. (PDF)
