# Supplementary material for: Assessment of community support for Wolbachia-mediated population suppression as a control method for Aedes aegypti mosquitoes in a community cohort in Puerto Rico
Source: PLoS Negl Trop Dis. 2021 Dec 6;15(12):e0009966. doi: 10.1371/journal.pntd.0009966 (PMC8675917; doi:10.1371/journal.pntd.0009966)
Supplement: S1 Appendix — (PDF) [file pntd.0009966.s001.pdf]

PID:

Acceptability of Potential Mosquito Control Activities

Introduction: In this section, we will discuss various methods to control *Aedes aegypti* mosquitos, which can transmit diseases such as dengue, Zika, and chikungunya. These mosquito control methods are used at different stages in the mosquito life cycle, so we will begin by explaining the mosquito life cycle:

***Interviewer**: Explain here the* Aedes aegypti *mosquito life cycle, using the visual aid card or the video of the Aedes aegypti mosquito life cycle. If using the visual aid card, read the script below.*

- It is a dark-colored mosquito, with white bands on its legs and body. It likes to be close to people, inside and outside houses.
- *Aedes aegypti* mosquitos bite mainly during daytime, although they will bite at night under artificial lighting.
- Only female mosquitos bite, they need to obtain blood to produce their eggs. Male mosquitoes don't bite.
- Female mosquitoes lay their eggs on the walls of any water-holding container, inside or outside homes.
- Eggs can survive up to 8 months stuck to the walls of containers where they are laid.
- When eggs come in contact with in water, larvae hatch.
- Larvae live in water where they feed and become pupae in as little as 5 days and stay near the surface where they breathe.
- After 2 to 3 days, pupae become adult flying mosquitoes.
- It can take just 7 to 10 days for an egg to become an adult mosquito.

Now we will talk about some methods used to control mosquitos. I will briefly explain each method. All of these methods have been used previously in the United States, Puerto Rico, or in other countries. I would like to know if you think these actions could be useful in your community. There are no right or wrong answers, I would just like to know your opinion.

1. Have you ever heard of **indoor residual spraying (IRS)**?

☐ Yes | ☐ No | ☐ Doesn't know | ☐ No response

***Interviewer:** Show the visual aid for IRS.*

Interviewer read the following:

- In this type of spraying, a licensed professional worker sprays the walls and other surfaces inside a house with a long-lasting insecticide, meaning that the insecticide continues working for several months.
- This way, the mosquitoes that rest on the sprayed surfaces, die.
- It might have a slight odor for several hours after applying it, but it does not cause harm to people when it is done correctly.
- It requires the permission and availability of a resident to enter the house to spray.
- To be effective in reducing number of mosquitoes, it needs to be applied to the majority of houses in an area.
- The spraying should be reapplied every 1 to 3 months to keep mosquito populations low.
- Over time, with repeated use, mosquitoes can become resistant to insecticides. Resistance means that the mosquitoes get used to the insecticide and do not die when they rest on sprayed surfaces.
- IRS has been used in many countries, including PR and USA.

1a. Would you support the use of **indoor residual spraying?**

***Interviewer**: Read the responses.*

☐ Support
☐ Neutral
☐ Oppose →
☐ No response

1b. What are the reasons you oppose?

***Interviewer:** Do not suggest responses. Choose all reasons mentioned below. Use "other" for reasons not listed.*

☐ Environmental impact
☐ Wild animal health
☐ Human health
☐ Pet health
☐ Don't think it is/would be effective in this community
☐ Not a community priority/arboviruses are not a concern here
☐ Strong smell
☐ Other: ______________________________

2. Have you ever heard of **AGO traps for mosquitoes**?

☐ Yes | ☐ No | ☐ Doesn't know | ☐ No response

Interviewer: *Show the visual aid for AGO traps.*

Interviewer read the following:

- AGOs have two components: a 5-galon black plastic container with water and hay, and a capture chamber that contains sticky paper and a metal screen.
- The water and hay, and the dark color of the trap, attract female mosquitoes that look for containers to lay their eggs.
- Once the female mosquito is inside the capture chamber of the trap, the screen prevents it reaching the water.
- When the mosquito rests on the walls of the capture chamber, it gets stuck to the sticky paper.
- No professionally trained staff are needed to install the trap.
- Traps require maintenance every 2 months to maintain efficacy and avoid becoming mosquito breeding sites.
- To reduce the number of mosquitoes, AGOs must be used in the majority of houses in a community.
- AGOs have been used in several places in Puerto Rico, including Caguas and Salinas, and have reduced the number of mosquitoes.

2a. Would you support the use of **AGO traps for mosquitoes**?

Interviewer: *Read the responses.*

☐ Support
☐ Neutral
☐ Oppose →
☐ No response

2b. What are the reasons you oppose?

Interviewer: *Do not suggest responses. Choose all reasons mentioned below. Use "other" for reasons not listed.*

☐ Environmental impact
☐ Wild animal health
☐ Human health
☐ Pet health
☐ Don't think it is/would be effective in this community
☐ Not a community priority/arboviruses are not a concern here
☐ If the traps are not maintained they can become mosquito breeding sites
☐ Other: _______________________________

Sterile Insect Technique

Interviewer read the following:

- Sterile Insect Technique is a method to control insects and has been used for many different insect species.
- The principle of the sterile insect technique involves releasing many sterile male insects to compete with wild fertile male insects.
- When a female mates with an introduced sterile male, there are no offspring and the population of the insects will decrease.
- In mosquitoes, there are different ways of making the males sterile. Examples include: genetic modification and introduction of a bacteria. Both of these are created in a lab.
- Large numbers (many thousands) of sterile mosquitoes need to be released periodically in order to keep the number of sterile males stable within a population.
- This technique can be used in many different insects and has been used primarily in agriculture.
- Each use of sterile insect technique is species-specific and does not pose a risk to humans or other animals or insects.

Now we will discuss two ways of using sterile insect technique in mosquitoes.

3. Have you ever heard of **male genetically modified (GM) mosquitoes**?

☐ Yes | ☐ No | ☐ Doesn't know | ☐ No response

Interviewer: *Show the visual aid for male GM mosquitoes.*

Interviewer read the following:

- In this method, male genetically modified mosquitoes are released. Female mosquitoes are not released.
- Genetically modified mosquitoes are mosquitoes that have had part of their genetic information changed in a laboratory.
- These male mosquitoes mate with wild females and pass to their offspring a gene that prevents larvae and pupae from developing normally. In this way, larvae and pupae die before they become adult mosquitoes.
- These mosquitoes need to be released several times per week for a prolonged period to keep the mosquito populations low.
- Mosquito population will eventually increase again when the releases of genetically modified mosquitoes stop.
- The use of genetically modified mosquitoes is safe for people, animals, and the environment.
- GM male mosquitoes have been used in Brasil, Panama, and Cayman Islands.
- At present, they have not been evaluated in the US, but permissions have been granted for evaluations to take place in a number of states in the US.

3a. Would you support the use of **male genetically modified mosquitoes**?

Interviewer: *Read the responses.*

☐ Support
☐ Neutral
☐ Oppose →
☐ No response

3b. What are the reasons you oppose?

Interviewer: *Do not suggest responses. Choose all reasons mentioned below. Use "other" for reasons not listed.*

☐ Environmental impact
☐ Wild animal health
☐ Human health
☐ Pet health
☐ Don't think it is/would be effective in this community
☐ Not a community priority/arboviruses are not a concern here
☐ Concerned about safety of use of GM mosquitoes
☐ GM mosquitoes could transmit their genes to other mosquitoes, people, or animals
☐ Other: ______________________________

4. Have you ever heard of mosquitoes with ***Wolbachia***?

☐ Yes | ☐ No | ☐ Doesn't know | ☐ No response

Interviewer read the following:

- Wolbachia is a bacterium that lives inside many insects, including some mosquito species.
- There is a type of Wolbachia that makes Aedes aegypti mosquitoes less capable of transmitting dengue, chikungunya, and Zika viruses to people.
- Since this type of Wolbachia has not been found in *Aedes aegypti* mosquitoes, scientists have introduced this bacterium in the mosquitoes in a lab, so that they are less capable of transmitting viruses.
- Wolbachia does not cause disease in humans and its use is safe for people, animals and the environment.

Mosquitoes with *Wolbachia* can be used in two different ways.

Interviewer: *Show the visual aid for male mosquitoes with Wolbachia.*

Interviewer read the following:

- This technique can be used releasing **only male** *Aedes aegypti* mosquitoes carrying Wolbachia. Female mosquitoes carrying Wolbachia are not released. When it is used this way, it is considered a sterile insect technique.
- When male mosquitoes carrying Wolbachia mate with wild females without Wolbachia.
- These female mosquitoes without *Wolbachia* lay their eggs, but these do not hatch.
- Los mosquitos deben liberarse varias veces a la semana a lo largo del tiempo para mantener bajas las poblaciones de mosquitos.
- These mosquitoes need to be released several times per week for a prolonged period to keep the mosquito populations low.
- Mosquito population will eventually increase again when the releases of mosquitoes carrying Wolbachia stop.
- Male mosquitoes with Wolbachia have been used in California and the Florida Keys and have been approved for evaluation in Miami. FL.

4a. ¿ Would you support the use of **male mosquitoes with *Wolbachia***?

Interviewer*: Read the responses.*

☐ Support
☐ Neutral
☐ Oppose →
☐ No response

4b. What are the reasons you oppose?

Interviewer: *Do not suggest responses. Choose all reasons mentioned below. Use "other" for reasons not listed.*

☐ Environmental impact
☐ Wild animal health
☐ Human health
☐ Pet health
☐ Don't think it is/would be effective in this community
☐ Not a community priority/arboviruses are not a concern here
☐ Concerned about safety of use of mosquitoes with *Wolbachia*
☐ Other: ______________________________

Interviewer: *Show the visual aid for male and female mosquitoes with Wolbachia.*

Interviewer read the following:

- There is another way to use Wolbachia, that is not a sterile insect technique, in which both **female and male** *Aedes aegypti* mosquitoes carrying *Wolbachia* are released.
- When the female mosquito carrying *Wolbachia* mates with a male mosquito <u>with or without</u> *Wolbachia*, the bacterium is passed through the female to its offspring from generation to generation.
- Over time, the amount of mosquitoes carrying *Wolbachia* increases and replaces the wild mosquitoes without *Wolbachia*.
- After releasing these mosquitoes many times over a period of months, the population of mosquitoes carrying *Wolbachia* will stay stable without the need for further releases.
- Mosquitoes carrying *Wolbachia* are less capable of disease transmission.
- There will still be mosquitoes in the community—the intention of this method is not the reduction of the number of mosquitoes but to reduce the risk of disease outbreaks.
- This technology has been used in Colombia, Brazil, and other countries. Currently, there are no established regulations for the use of both male and female mosquitoes carrying *Wolbachia* in the US.

5a. Would you support the use of **male and female mosquitoes with *Wolbachia***?

Interviewer*: Read the responses.*

☐ Support
☐ Neutral
☐ Oppose →
☐ No response

5b. What are the reasons you oppose?

Interviewer: *Do not suggest responses. Choose all reasons mentioned below. Use "other" for reasons not listed.*

☐ Environmental impact
☐ Wild animal health
☐ Human health
☐ Pet health
☐ Don't think it is/would be effective in this community
☐ Not a community priority/arboviruses are not a concern here
☐ Concerned about safety of use of mosquitoes with *Wolbachia*
☐ It doesn't make sense because we will still have mosquitoes
☐ Other: ______________________________

6. Have you ever heard of **larvicides**?

☐ Yes | ☐ No | ☐ Doesn't know | ☐ No

Interviewer: *Show the visual aid for larvicides.*

Interviewer read the following:

- Larvicides are insecticides that kill mosquito larvae. If applied correctly, larvicides can potentially reduce the number of adult mosquitoes.
- There are different types of larvicides and they come in different formulations, for example: liquid, granules and briquettes. Liquid larvicides can be applied using truck mounted equipment, spraying them over houses, vegetation, empty lots and other places. In this way, this method may be effective to treat most of the places where larvae are found.
- Larvicides can also be applied manually to water where larvae are found, for example, fountains, tree holes, gutters, etc.
- When larvicide is used according to specific label instructions, it does not affect the environment, people, or pets.
- Larvicide application needs to be repeated according to specific label instructions.
- Although unlikely, with repeated use, larvae could become resistant to the effects of larvicides. Resistance means that the larvae resists the effects of the larvicide and don't die.
- Larvicides are used around the world.

6a. Would you support the use of **larvicides**?

Interviewer: *Read the responses.*

☐ Support
☐ Neutral
☐ Oppose →
☐ No response

6b. What are the reasons you oppose?

Interviewer: *Do not suggest responses. Choose all reasons mentioned below. Use "other" for reasons not listed.*

☐ Environmental impact
☐ Wild animal health
☐ Human health
☐ Pet health
☐ Don't think it is/would be effective in this community
☐ Not a community priority/arboviruses are not a concern here
☐ Concerned about safety of use of larvicides
☐ Other: ______________________________
